# The rootstock modifies the arbuscular mycorrhizal community of the root system, while the influence of the scion is limited in grapevines

**DOI:** 10.1111/1758-2229.13318

**Published:** 2024-08-22

**Authors:** Vincent Lailheugue, Romain Darriaut, Joseph Tran, Marine Morel, Elisa Marguerit, Virginie Lauvergeat

**Affiliations:** ^1^ EGFV, Univ. Bordeaux, Bordeaux Sciences Agro, INRAE, ISVV Villenave d'Ornon France; ^2^ Present address: Univ Rennes, CNRS, ECOBIO (Ecosystèmes, biodiversité, évolution)—UMR 6553 Rennes France

## Abstract

Understanding the effects of grapevine rootstock and scion genotypes on arbuscular mycorrhizal fungi (AMF), as well as the roles of these fungi in plant development, could provide new avenues for adapting viticulture to climate change and reducing agrochemical inputs. The root colonization of 10 rootstock/scion combinations was studied using microscopy and metabarcoding approaches and linked to plant development phenotypes. The AMF communities were analysed using 18S rRNA gene sequencing. The 28S rRNA gene was also sequenced for some combinations to evaluate whether the method changed the results. Root colonization indexes measured by microscopy were not significantly different between genotypes. Metabarcoding analyses showed an effect of the rootstock genotype on the β‐diversity and the enrichment of several taxa with both target genes, as well as an effect on the Chao1 index with the 18S rRNA gene. We confirm that rootstocks recruit different AMF communities when subjected to the same pedoclimatic conditions, while the scion has little or no effect. Significant correlations were observed between AMF community composition and grapevine development, suggesting that AMF have a positive effect on plant growth. Given these results, it will be important to define consensus methods for studying the role of these beneficial micro‐organisms in vineyards.

## Graphical abstract

The grapevine rootstock is a major player in the adaptation of viticulture to agroecology. Their ability to associate with arbuscular mycorrhizal fungi can be one of the key processes to integrate into selection programs.

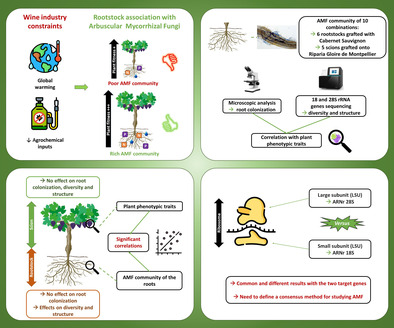

## INTRODUCTION

Nowadays, about 80% of all plant species form intimate symbiotic associations with a class of ubiquitous soil microorganisms called arbuscular mycorrhizal fungi (AMF) in a wide range of terrestrial environments (Smith & Read, 2008). Fossilized AMF‐type hyphae and spores from the Ordovician period suggest that arbuscular mycorrhizae (AM) symbiosis appeared 460 million years ago and played a key step in the evolution of terrestrial plants (Redecker et al., 2000). AMF are obligate biotrophs which provide mineral nutrients (mainly phosphorus [P] and sometimes nitrogen [N]) to the host plant in exchange of 4% to 23% of photosynthetically fixed carbon (Salmeron‐Santiago et al., 2021). The establishment of AM symbiosis is a dynamic process composed of distinct stages: presymbiotic communication, contact and penetration, outer cortex invasion, arbuscule formation, and vesicle and spore formation (Choi et al., 2018). This association is achieved by chemical communication between both partners in the rhizosphere, defined as the area of soil proximal to the roots. AMF release *Myc* factors that are perceived by plant receptors to initiate a signalling pathway. Strigolactones (SLs) are then exuded from the roots and induce the germination of AMF spores, as well as the growth of hyphae until they reach the root surface to form hyphopodia. The epidermal cells of plant roots undergo a series of reprogramming events to form a prepenetration apparatus that guides the hyphae through the root epidermal cells. Hyphae develop in the cortical cells of the root, forming highly branched structures called arbuscules, surrounded by a periarbuscular membrane that provides a wide interface for the exchange of nutrients (Wang et al., 2017).

Around 300 AMF species have been described to date (Alrajhei et al., 2022). Despite the controversy that still surrounds AMF phylogeny, Redecker et al. (2013) proposed a consensus classification of the Glomeromycetes division into four orders (*Diversisporales*, *Glomerales*, *Archaeosporales* and *Paraglomerales*) subdivided into 11 families. Quantification of the AMF community in soil and roots has traditionally been performed using microscopic methods such as spore density (Gerdemann & Nicolson, 1963) and root colonization (Phillips & Hayman, 1970). Alternatively, lipid biomarker analysis can be used to measure AMF population density (Sharma & Buyer, 2015). Very recently, molecular methods have been developed to estimate mycorrhization intensity by qPCR (Bodenhausen et al., 2021) and RT‐qPCR (Duret et al., 2022). Finally, sequencing of AMF ribosomal Internal Transcribed Spacers (ITS), 18S rRNA or 28S rRNA genes provided deeper insights into AMF diversity and structures (Darriaut et al., 2023; Suzuki et al., 2020).

Over the past decade, several authors have proposed that AMF can have beneficial effects on viticultural adaptation to sustainable agriculture and climate change (Darriaut et al., 2022; Torres et al., 2018; Trouvelot et al., 2015). These fungi are known to improve grapevine mineral uptake by increasing the soil exploration surface and activating plant transport proteins for P, N and other elements (Moukarzel et al., 2023; Schreiner, 2005; Trouvelot et al., 2015). Moreover, AMF increase grapevine tolerance to biotic stresses such as diseases caused by pathogenic fungi (Cruz‐Silva et al., 2021; Moukarzel et al., 2022) and viruses (Gaši et al., 2023). They have also been reported to play several roles in mitigating abiotic stresses in grapevines such as water stress, drought, and copper toxicity (Aguilera et al., 2022; Brunetto et al., 2023; Nogales et al., 2021). AMF improve the structural stability and quality of the soil (Trouvelot et al., 2015). Finally, studies have suggested that mycorrhizal symbiosis can improve berry quality (Goicoechea et al., 2023; Torres et al., 2018). In this context, finding rootstock or scion genotypes favouring association with AMF, as well as the discovery of new AMF species better adapted to winegrowing terroirs, could provide a means of adapting to the challenges facing the wine industry.

Since the phylloxera crisis, *V. vinifera* has been cultivated and grafted onto hybrid American *Vitis* rootstocks that are naturally tolerant to soil‐born aphids. Many signalling and regulating molecules, such as hormones, are exchanged between partners (Ollat et al., 2017). Darriaut et al. (2022) showed that the *Vitis riparia* Gloire de Montpellier (RGM) rootstock presented a significantly higher intensity of mycorrhization, as well as a higher relative abundance of *Glomeromycota* sequences than *Vitis berlandieri x rupestris* hybrid 1103Paulsen (1103P) when grown in a symptomatic vineyard soil. These results are consistent with those of Cochetel et al. (2018) which suggest that RGM produces more strigolactones‐like compounds than 1103P when subjected to low nitrogen availability. A recent study confirmed that RGM and 1103P produced a different pattern of SLs in response to nitrogen starvation (Lailheugue et al., 2023). Other studies have explored the ability of grapevine rootstocks to associate with AMF. Moukarzel et al. (2021) showed the effect of the rootstock genotype on the AMF community composition based on denaturing gel electrophoresis (DGGE) and trap cultures. However, no effects of rootstock genotypes were observed on the α‐ and β‐diversity metrics among the factors studied by Fors et al. (2023). We recently investigated the composition of the rhizosphere and the root endosphere microbiomes of 10 combinations of scion/rootstock grafted grapevines sampled in the same experimental vineyard (Lailheugue et al., 2024). The root samples (rhizosphere and root endosphere) were harvested in May 2021 and the *Glomeromycota* community was analysed using Illumina sequencing of the *Glomeromycota* 28S rRNA gene. We observed that the rootstock genotype had an impact on AMF richness and diversity in the root endosphere, as well as on the Bray–Curtis index in both components of the root system. The scion did not significantly influence the AMF community of the root system.

As the effect of rootstock and scion genotypes on AMF remains poorly documented, we took new root samples, this time in September of the same year, to take a closer look at the mycorrhizal association. Young rootlets were stained to quantify root colonization in the 10 rootstock scion combinations (six rootstocks grafted on a Cabernet‐Sauvignon (CS) scion and five scions grafted on RGM rootstock). The AMF community was analysed by sequencing the glomeromycotan 18S rRNA gene in all the combinations. At the same time, to compare the effect of the target gene in AMF analyses based on metabarcoding approaches, the glomeromycotan 28S rRNA gene was sequenced for the six rootstocks grafted with CS. Finally, the variables used to characterize the AMF community were correlated with plant phenotypic traits.

## EXPERIMENTAL PROCEDURES

### Site, plant material, sample collection and plant phenotyping

The experiment was conducted in 2021 in the GreffAdapt plot (Lailheugue et al., 2024; Marguerit et al., 2019), located near Bordeaux, France (44°47′26.6″N 0°34′26.7″W). It consists in 55 *Vitis* sp. rootstock genotypes grafted with five *Vitis vinifera* scions, and 15 plants per combination divided in three blocks. The plot has been planted in 2015 with a density of 6250 vines/ha. Aurea Agrosciences (Orléans, France) analysed the soil and classified it as sandy and gravelly (Table S1). The climate is oceanic, with mild winters and high rainfall. The plot is not irrigated. Vines are pruned using the simple Guyot method and trained with a vertical trellis system. The inter‐rows are not tilled, but the area under the vines is tilled mechanically. Weeds (mainly ray grass) growing on the inter‐rows are mowed when necessary. Fungicide treatments are applied regularly to limit the development of downy and powdery mildew during the vegetative period.

To study the ability of different rootstock genotypes to associate with AMF, six rootstock genotypes were chosen based on their different genetic origins, contrasting drought tolerance and vegetative vigour conferred to the scion: 1103 Paulsen (1103P), 3309 Couderc (3309C), 41 B Millardet et de Grasset (41 B), Nemadex Alain Bouquet (Nem), Riparia Gloire de Montpellier (RGM) and Selection Oppenheim 4 (SO4) (Table S2). All of them were grafted with Cabernet‐Sauvignon scion. The RGM rootstock grafted with Grenache (Gre), Pinot Noir (PN), Syrah (Syr) and Ugni Blanc (UB) was selected to evaluate the effect of the scion genotype on AMF association. Root samples were collected in September 2021 (when the plants were at the main stage 8 (berry ripening) and sub‐stage 8.9 (the berries are ripe for harvesting) according to the BBCH classification), on five plants per scion/rootstock combination, grown on the block 1 of the plot. Roots were taken at a depth of 20–30 cm, at three points located between 10 and 30 cm from the trunk of the plant. For microscopic analyses, the smallest, young, white roots were collected in a Falcon tube containing 50 ml of 10% ethanol and refrigerated to 4°C before being processed. For metabarcoding analyses, roots of all sizes were rinsed with sterile water, transferred in empty tubes, frozen using liquid nitrogen and stored at −80°C before DNA extraction.

Phenotypic monitoring was carried out on the vines in 2021. This included the number of shoots, fertility (number of bunches per shoot), winter pruning weight and the conferred vigour (pruning weight / number of shoots). The number of bunches, berry yield and δ^13^C of berry juice were assessed at the harvest, when the berries from the different scions were at maturity (between the 2nd of September for Pinot Noir and the 7th of October for Cabernet Sauvignon). δ^13^C values were measured by ISC SILVATECH (France).

### Staining and quantification of root mycorrhiza by microscopy

The staining protocol was adapted from the method developed by Phillips and Hayman (1970). This involves incubating the roots in 40 ml KOH 10% for 30 min at 95°C. After this step, 500 μl of H_2_O_2_ 30% were added and left to react for 5 min at room temperature. The roots were then rinsed three times with distilled water, drained and transferred to the staining solution (5% of Sheaffer black ink 94231 and 8% of FLUKA acetic acid 4574) for 5 min at 90°C. The roots were rinsed three times with distilled water, incubated in 8% of acetic acid at room temperature for 15 min and rinsed one last time with distilled water. The mycorrhization rate was estimated using the method of Trouvelot et al. (1986), with a scale of six classes graded from 0 to 5 based on the level of mycelium present in each root fragment. Thirty root fragments of 1 cm long per plant were observed by microscopy (LEICA DM750) to assess the mycorrhization frequency (F%), the overall mycorrhization intensity (M%) and the mycorrhization intensity of mycorrhized fragments (m%), calculated using the Mycocalc program (INRAe, Dijon).

### DNA extraction from the roots

The roots were ground in 35 ml stainless‐steel grinding jars with 20 mm stainless‐steel balls at 30 oscillations per second for 30 s with the mixer mill MM400 (Retsch, Haan, Germany), frozen using liquid nitrogen and then stored at −80°C until processing. DNA was extracted from 150 mg of frozen root powder using the protocol of Berger et al. (2022). Extracted DNA was quantified on a Qubit 3.0 fluorometer (Thermo Fisher Scientific) using the Qubit dsDNA HS Assay Kit, and its quality was checked with a NanoDrop 2000/2000c spectrophotometer (Thermo Fisher Scientific). The DNA was then stored at −20°C until further use.

### Amplification of the glomeromycotan 18S and 28S rRNA genes

The glomeromycotan 18S rRNA gene was amplified by PCR using the primer pair AMV4.5NF/AMDGR (Suzuki et al., 2020) while the glomeromycotan 28S rRNA gene was amplified using a nested PCR initiated with the primer pair LR1/NDL22. The product was diluted 100‐fold and used as a template for the second PCR using the primer pair FLR3/FLR4 to amplify the glomeromycotan 28S rRNA gene (Lailheugue et al., 2024). All the PCR reactions were monitored in a final volume of 25 μl composed of 5 μl of 5× GoTaq colourless reaction buffer (Promega, France), 0.5 μl of each primer (10 μM), 0.5 μl of dNTPs (10 mM), 0.125 μl of GoTaq G2 DNA Polymerase (Promega, France), 1 μl of DNA extracted (1 ng μl^−1^) or 5 μl of the PCR product, amended with nuclease‐free water. The sequence of primers used, and the cycling condition are presented in Table S3. Further steps were carried out at the Plateforme Génome Transcriptome de Bordeaux (Cestas, France). The PCR products were purified with the platform‐specific SPRI magnetic beads (1× ratio) and quantified using Quant‐iT dsDNA assay kit (ThermoFisher, France). MID and Illumina sequencing adapters were added. Libraries were pooled in equimolar amounts using a Hamilton Microlab STAR robot and sequenced on an Illumina MiSeq platform using the MiSeq Reagent Nano Kit V2 (2 × 250 bp).

### Metabarcoding data processing

Obtained sequences were demultiplexed with index search at the PGTB facility. The quality of the sequences obtained were first checked with FastQC v.0.11.8 (Andrews, 2010). Sequences were quality filtered, trimmed, denoised, and clustered into Operational Taxonomy Units (OTUs) using the Galaxy‐supported pipeline FROGS (Escudié et al., 2018). This involved assembling raw forward and reverse reads for each sample into paired‐ended reads with a minimum overlapping of 50 nucleotides and 0.1 mismatch using the VSEARCH tool (Rognes et al., 2016). Primers were removed using Cutadapt (Martin, 2011), chimeras were detected and removed with UCHIME (Edgar et al., 2011), and clustering was performed using SWARM (Mahé et al., 2014) in the FROGS pipeline. The minimum sequence abundance proportion was set at 5e^−5^, with a minimum prevalence set at 4 to keep OTUs. Taxonomic assignments of 18S and 28S rRNA genes were performed against the MaarjAM database (Öpik et al., 2010). For the 28S rRNA gene, the sequences affiliated to “New_clade” at the class level were removed and one sample containing <5000 sequences was excluded before rarefying to the number of sequences in the sample containing the fewest sequences using the phyloseq “rarefy_even_depth” function. For the 18S rRNA gene, the “#N/A” sequences at the phylum level were removed and one sample containing <5000 sequences was excluded before rarefying.

### Statistical analyses

All the statistical analyses were performed on R (v4.2.1) using RStudio (2022.07.1). Figures were generated with *ggplot2* (v3.4.1) and *ggthemes* (v4.0.4) and arranged with *ggpubr* (v0.4.0). Chao1 and Simpson indexes were calculated using the “estimate_richness” function, and PCoA analyses based on the Bray–Curtis distance were performed using the “plot_ordination” function, both from *phyloseq* (1.38.0) (McMurdie & Holmes, 2013). Linear Discriminant Analysis Effect Size (LEfSe) was carried out using the “run_lefse” function from *microbiomeMarker* (1.2.2) with data transformation (log10), lda_cutoff set at 4, kw_cutoff and wilcoxon_cutoff at 0.05 (Cao et al., 2022). The effects of the genotypes on the α‐diversity metrics and the variables measured by microscopy were tested with One‐Way ANOVA or Kruskal‐Wallis test, depending on whether the assumptions of the parametric tests were met. Normality and heteroscedasticity were checked on residuals with Shapiro and Bartlett tests, respectively. Comparisons between genotypes were performed using “pairwise.t.test” or “pairwise.wilcox.test” functions with Bonferroni correction from *stats* (4.2.1). To test the effect of the genotype on the Bray–Curtis index, Permutational Analysis of Variance (PERMANOVA) with 999 permutations was carried out with the “adonis2” function from *vegan* (2.6.4). Genotypes were then compared together using “pairwise.adonis2” from *pairwiseAdonis* (0.4.1). Venn diagrams showing common and exclusive OTUs were generated with the following tool: http://bioinformatics.psb.ugent.be/webtools/Venn/. Correlation matrices were established using “ggcorrplot” from *ggcorplot* (0.1.4) with the “square” method based on *p* values calculated using “cor.mtest.”

## RESULTS

### Microscopic analysis after root staining did not reveal significant differences in terms of the mycorrhization rate depending on the genotypes of the rootstock and the scion

**FIGURE 1:**
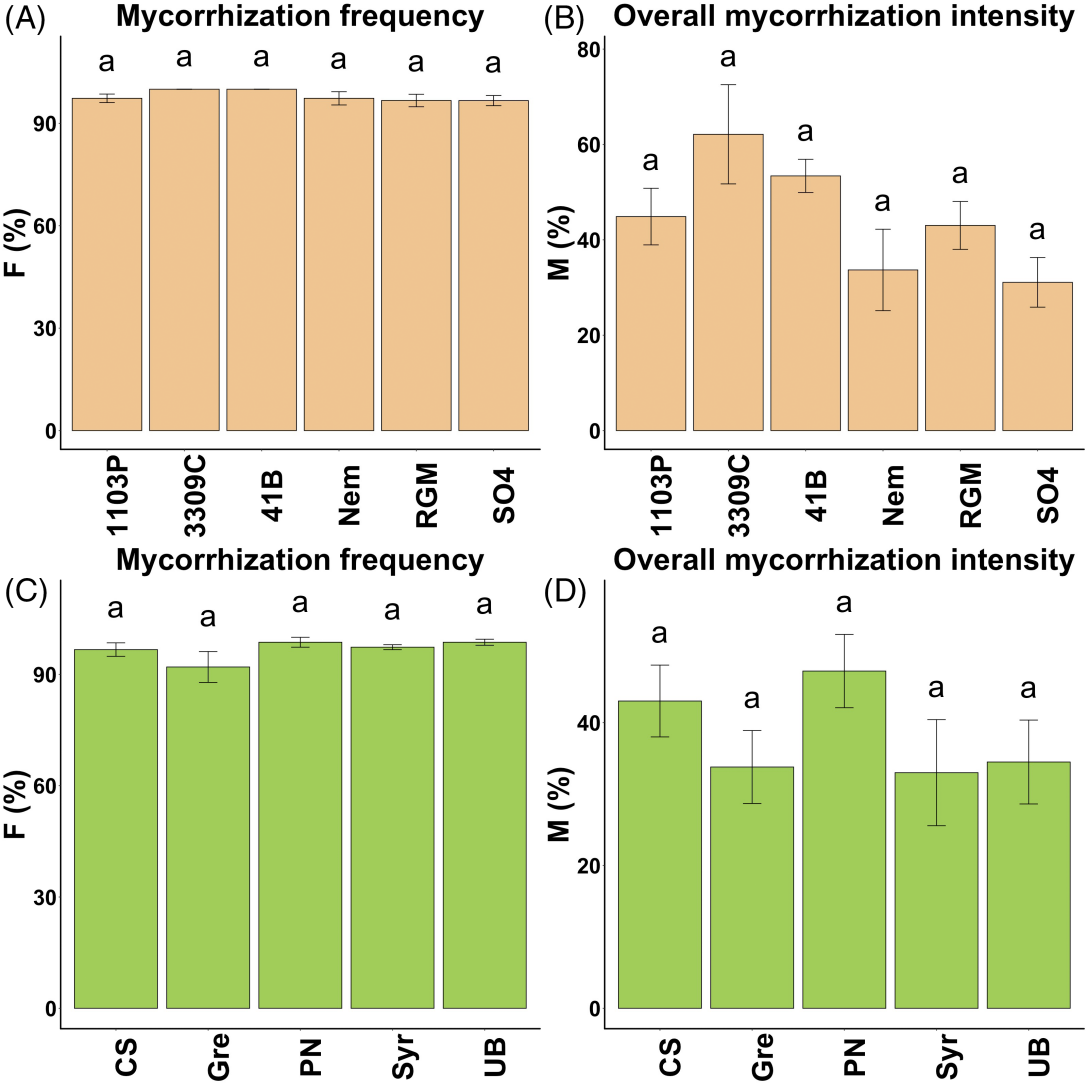
Comparison of the mycorrhization frequency (F%) and the overall mycorrhization intensity (M%) between genotypes of rootstock grafted with Vv cv. Cabernet Sauvignon (A, B) or scion grafted on RGM (C, D). Data are represented as mean ± SE (*n* = 5). Letters indicate significant differences between genotypes using the pairwise t‐test with Bonferroni correction (*p* value <0.05).

The quantification of the mycorrhization frequency (F%) showed that all the plants studied were naturally mycorrhized in the GreffAdapt plot (Figure 1). ANOVA showed no effect of the rootstock genotype on the mycorrhization frequency (F%) (Table 1 and Figure 1). The rootstock had an effect on the overall mycorrhization intensity (M%) (Table 1), but no significant differences were observed between groups using the pairwise *t*‐test with Bonferroni correction (Figure 1B). The same result was obtained for the mycorrhization intensity of mycorrhized fragments (m%) (Table 1). When the five scions grafted on RGM were studied, no effect of the scion was reported on either parameter (F%, M%, and m%), suggesting that it did not contribute to the mycorrhization capacity of the root system (Table 1 and Figure 1).

**TABLE 1:** Effect of the genotype of rootstock or scion on root endomycorrhization, assessed by measuring the mycorrhization frequency (F%), the overall mycorrhization intensity (M%) and the mycorrhization intensity of mycorrhized fragments (m%).

	Rootstock			Scion		
	*p* value		PVE (%)	*p* value		PVE (%)
F%	0.272	ns	/	0.212	ns	/
M%	**0.032**	*	**38**	0.329	ns	/
m%	**0.039**	*	**37**	0.328	ns	/

*Note*: *p* values were calculated with One‐Way ANOVA and were considered significant (in bold) when the *p* value <0.05 (*n* = 5).

Abbreviations: PVE, percentage of variance explained.

### Comparison of rootstock AMF communities using 28S rRNA gene sequencing

**FIGURE 2:**
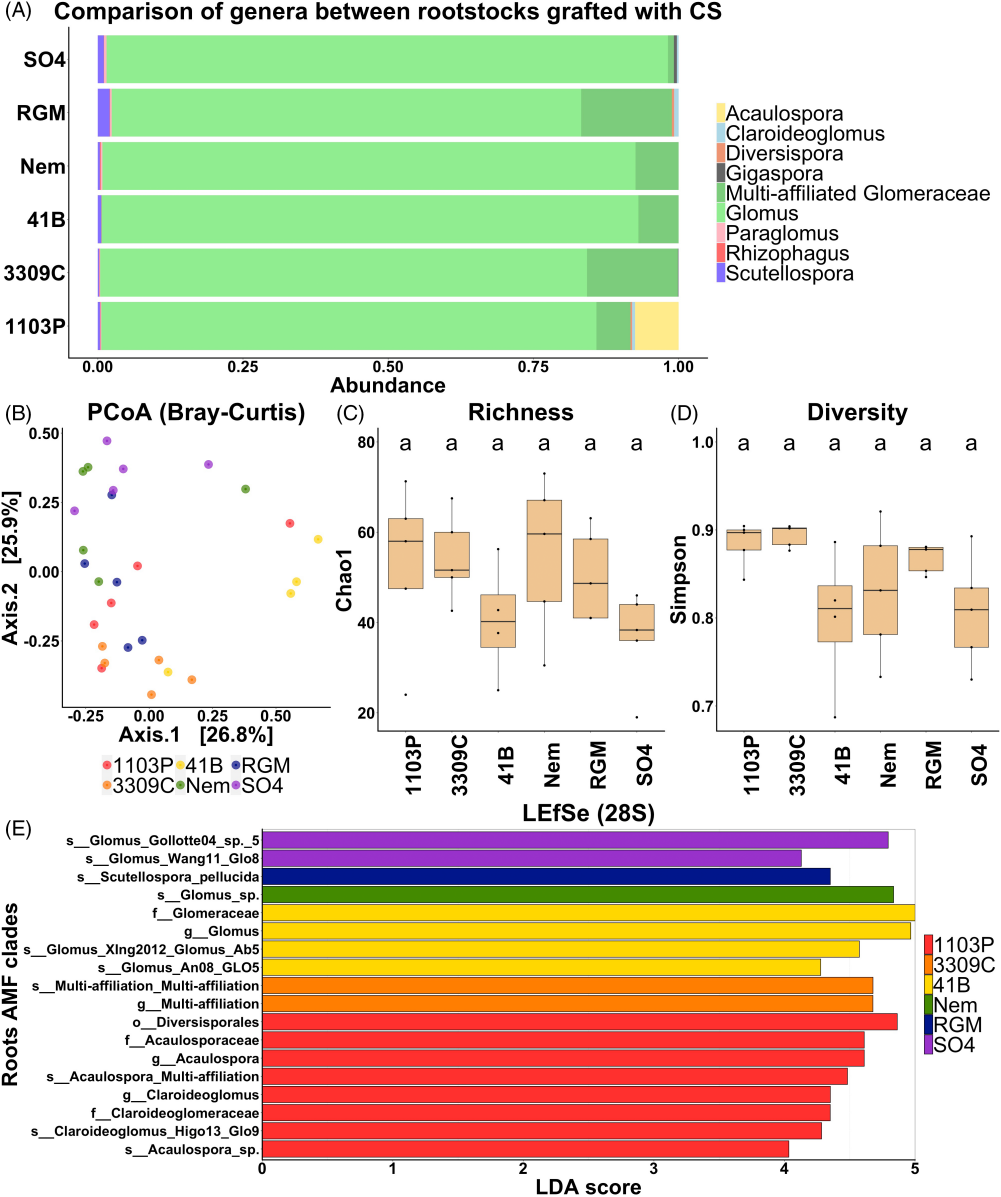
Comparison of AMF communities among the six rootstock genotypes grafted with CS and assessed with the 28S rRNA gene. Relative abundance of AMF genera (A). Principal coordinate analysis (PCoA) based on Bray–Curtis dissimilarity matrices coloured by the rootstock genotype showing the overall distribution pattern of AMF communities (B). AMF richness, represented by Chao1 (C) and diversity, represented by Simpson's index (D). *p* values were calculated using pairwise‐Student *t* tests with Bonferroni correction and were considered significant when the adjusted *p* value <0.05 (*n* = 5). Histogram of the linear discriminant analysis (LDA) scores revealed the most differentially abundant taxa among AMF communities (E).

The sequencing of the 28S rRNA gene showed dissimilar proportions of AMF genera between rootstock genotypes (Figure 2A). *Glomus* was the main genera in the roots among all genotypes (ranging from 81% to 97% of relative abundance). Several genera were genotype‐exclusive, such as *Acaulospora* in 1103P (representing 7.5% of abundance) or *Gigaspora* in SO4 (representing 0.5% of abundance). Nineteen OTUs were common between all the genotypes but several were exclusive to certain genotypes (six OTUs in 1103P, one in SO4, one in Nem and one in 41B) (Figure S1). An effect of the genotype was reported on the Bray Curtis index, explaining 47% of the variance (PVE) (Table 2). The Bray–Curtis index of SO4 was significantly different from those of 1103P and 3309C (Table S4). PCoA analyses based on this index did not show a clear genotype‐dependent clustering of individuals (Figure 2B). No effect of the rootstock genotype was observed on either of the α‐diversity metrics measured (Chao1 for richness and Simpson for diversity; Table 2 and Figure 2C/D). LEfSe analyses showed enrichments of AMF taxa in both genotype (1103P: one order, two genera, two families and two species; 3309C: one genus and one species; 41B: one genus, one family and two species; Nem: two species; RGM: one species; SO4: two species) (Figure 2E). Although α‐diversity metrics were not influenced by rootstock genotypes, these results suggest the potential involvement of the rootstock in structuring the AMF community of the root system.

**TABLE 2:** Influence of the rootstock and scion genotypes on α‐diversity (richness = Chao1, diversity = Simpson) and β‐diversity metrics (Bray–Curtis dissimilarity).

Gene	Factor	Chao1			Simpson			Bray–Curtis		
		*p* value		PVE (%)	*p* value		PVE	*p* value		PVE (%)
28S rRNA	Rootstock	0.209	ns	/	0.065	ns	/	**0.001**	***	**47**
18S rRNA	Rootstock	**0.020**	*	**46**	0.574	ns	/	**0.001**	***	**39**
	Scion	0.577	ns	/	0.349	ns	/	**0.016**	*	**31**

*Note*: *p* values were calculated with One‐Way ANOVA and were considered significant (in bold) when the *p* value <0.05 (*n* = 5).

Abbreviation: PVE, percentage of variance explained.

### Comparison of the AMF community between rootstock and scion genotypes by 18S rRNA gene sequencing

**FIGURE 3:**
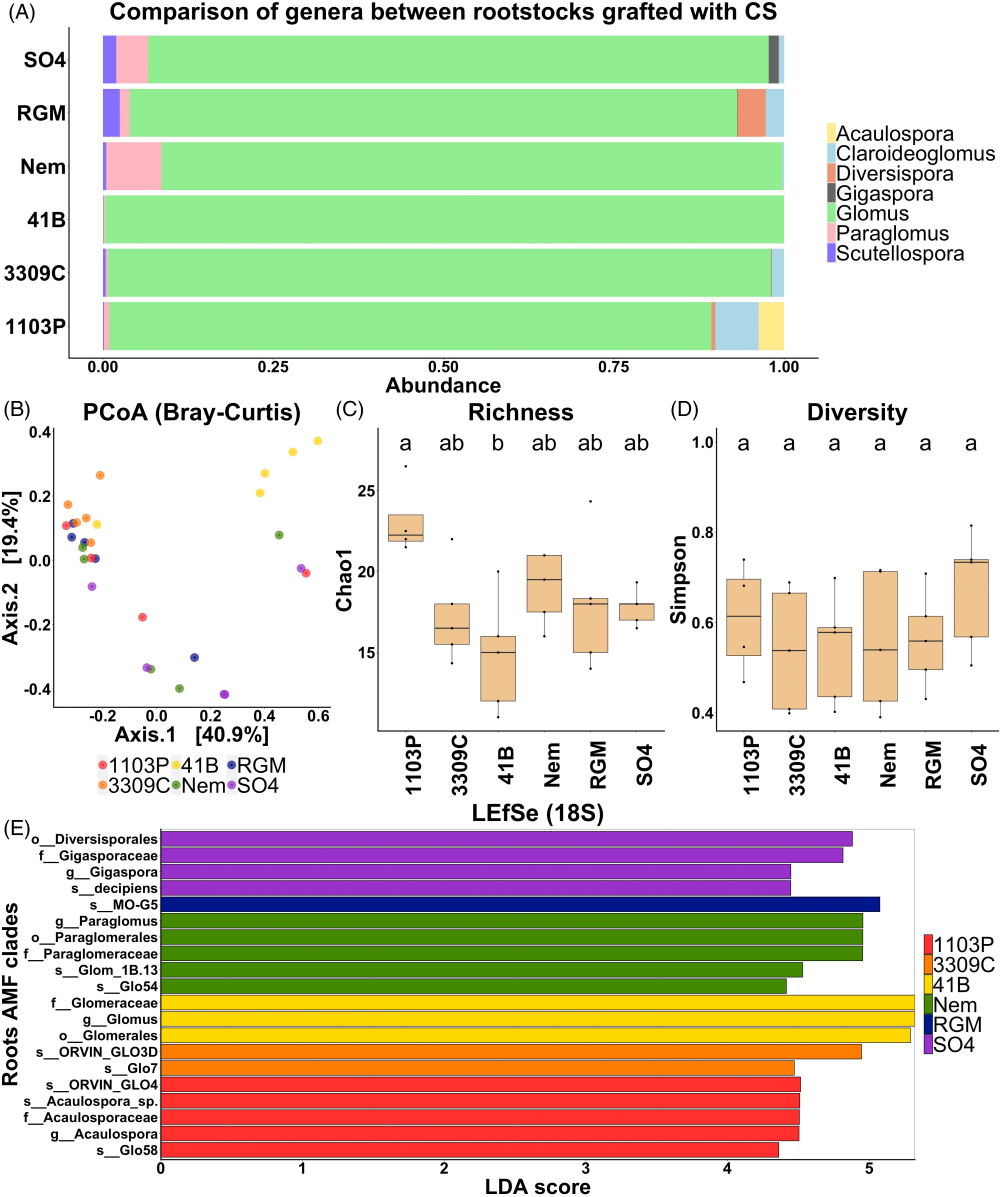
Comparison of AMF communities among the six rootstock genotypes grafted with CS and assessed with the 18S rRNA gene. Relative abundance of AMF genera (A). Principal coordinate analysis (PCoA) based on Bray–Curtis dissimilarity matrices coloured by the rootstock genotype showing the overall distribution pattern of AMF communities (B). AMF richness, represented by Chao1 (C) and diversity, represented by Simpson's index (D). *p* values were calculated using pairwise‐Student t tests with Bonferroni correction and were considered significant when the adjusted *p* value <0.05 (*n* = 5). Histogram of the linear discriminant analysis (LDA) scores revealed the most differentially abundant taxa among AMF communities (E).

The sequencing of the 18S rRNA gene also showed different proportions of AMF genera between rootstock genotypes (Figure 3A). The *Glomus* genus remained predominant in the roots (ranging from 88 to 99%). Only nine OTUs were common between all the rootstock genotypes, but two OTUs were specific to 3309C, one to 41B and one to Nem (Figure S2A). The rootstock genotype had an impact on the Bray–Curtis index, explaining 39% of the variance (Table 2). However, after the adjustment of the *p* value, the Bray–Curtis index was significantly different between SO4 and 3309C only (Table S5). PCoA analysis showed no genotype with a clear distinct AMF community structure (Figure 3B). Concerning α‐diversity, the rootstock genotype influenced the richness only, explaining 46% of the variance (Table 2). Indeed, the Chao1 index was significantly higher in 1103P than in 41B (Figure 3C). LEfSe analyses show enrichments of several taxa in both genotypes (1103P: one family, one genus and three species; 3309C: two species; 41B: one order, one family and one genus; Nem: one order, one family, one genus and two species; RGM: one species; SO4: one order, one family, one genus and one species) (Figure 3E). These results confirm that the rootstock genotype drives the root AMF community.

**FIGURE 4:**
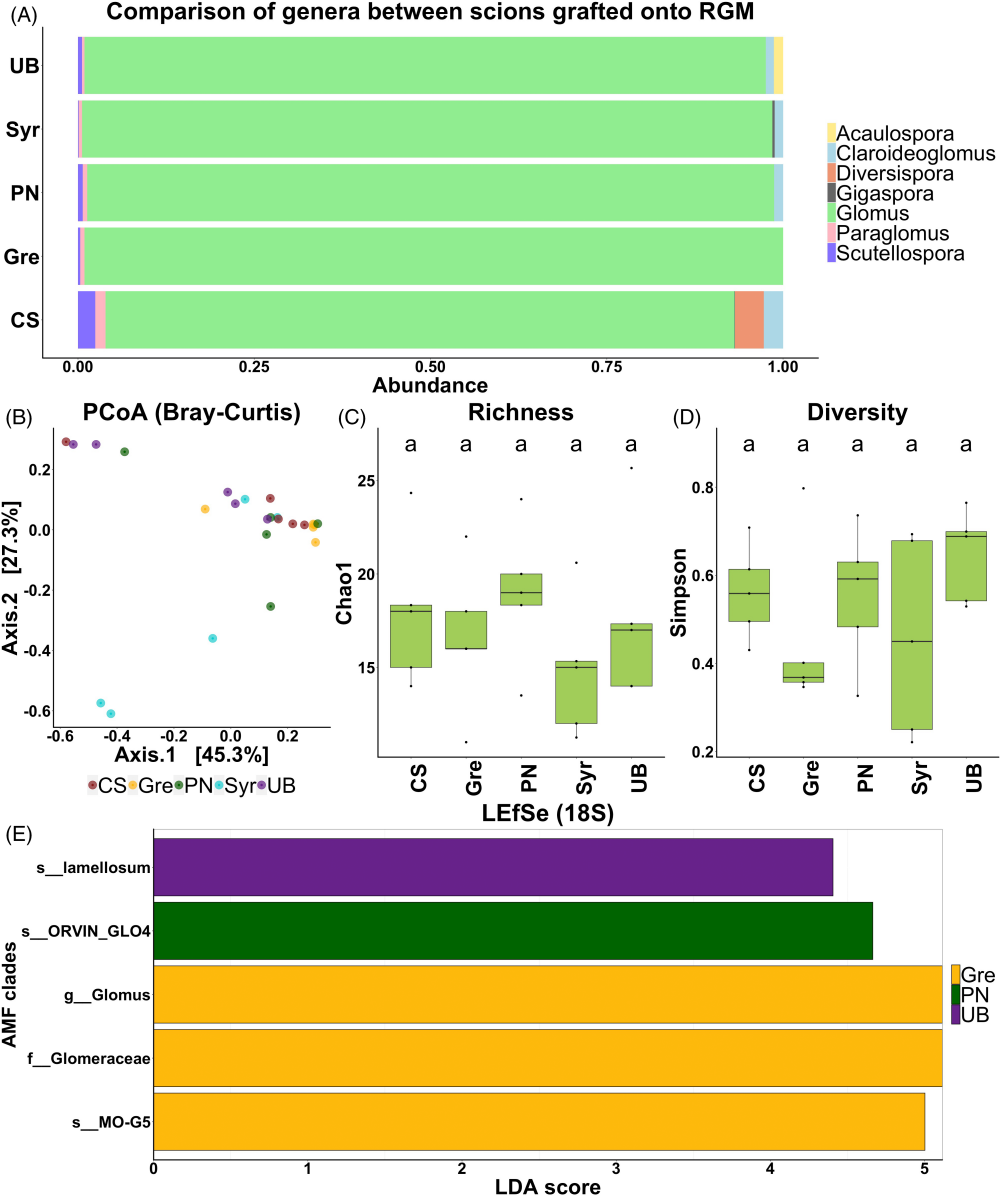
Comparison of AMF communities among the five scion genotypes grafted onto RGM and assessed with the 18S rRNA gene. Relative abundance of AMF genera (A). Principal coordinate analysis (PCoA) based on Bray‐Curtis dissimilarity matrices colored by the scion genotype showing the overall distribution pattern of AMF communities (B). AMF richness, represented by Chao1 (C) and diversity, represented by Simpson's index (D). *p* value were calculated using pairwise‐Student t tests with Bonferroni correction and were considered significant when the adjusted *p* value < 0.05. Histogram of the linear discriminant analysis (LDA) scores revealed the most differentially abundant taxa among AMF communities (E).

When the AMF communities of the five scions grafted on RGM were studied with the same methodology, slight dissimilarities were observed in the abundance of genera between genotypes. *Acaulospora* genera was specific to the root system of RGM grafted with UB, and *Diversispora* to those of RGM grafted with CS (Figure 4A). Fourteen OTUs were common between all the scion genotypes grafted onto RGM and five were specific to UB, three to Syrah, four to CS and four to PN (Figure S2B). Although the scion had an impact on the Bray–Curtis index (explaining 31% of variance), no significant difference was found between the genotypes (Table S6). This is consistent with the PCoA analysis showing no genotype‐dependent clustering (Figure 4B). Moreover, both ⍺‐diversity metrics were not influenced by the scion genotype (Table 2 and Figure 4C/D). LEfSe analyses show the enrichment of one family, one genus and one species in Grenache, one species in PN and one species in UB (Figure 4E). All these results suggest that the scion genotype contributes little or nothing to the structuring of the root AMF community.

### Influence of the AMF community on grapevine phenotypic traits

**FIGURE 5:**
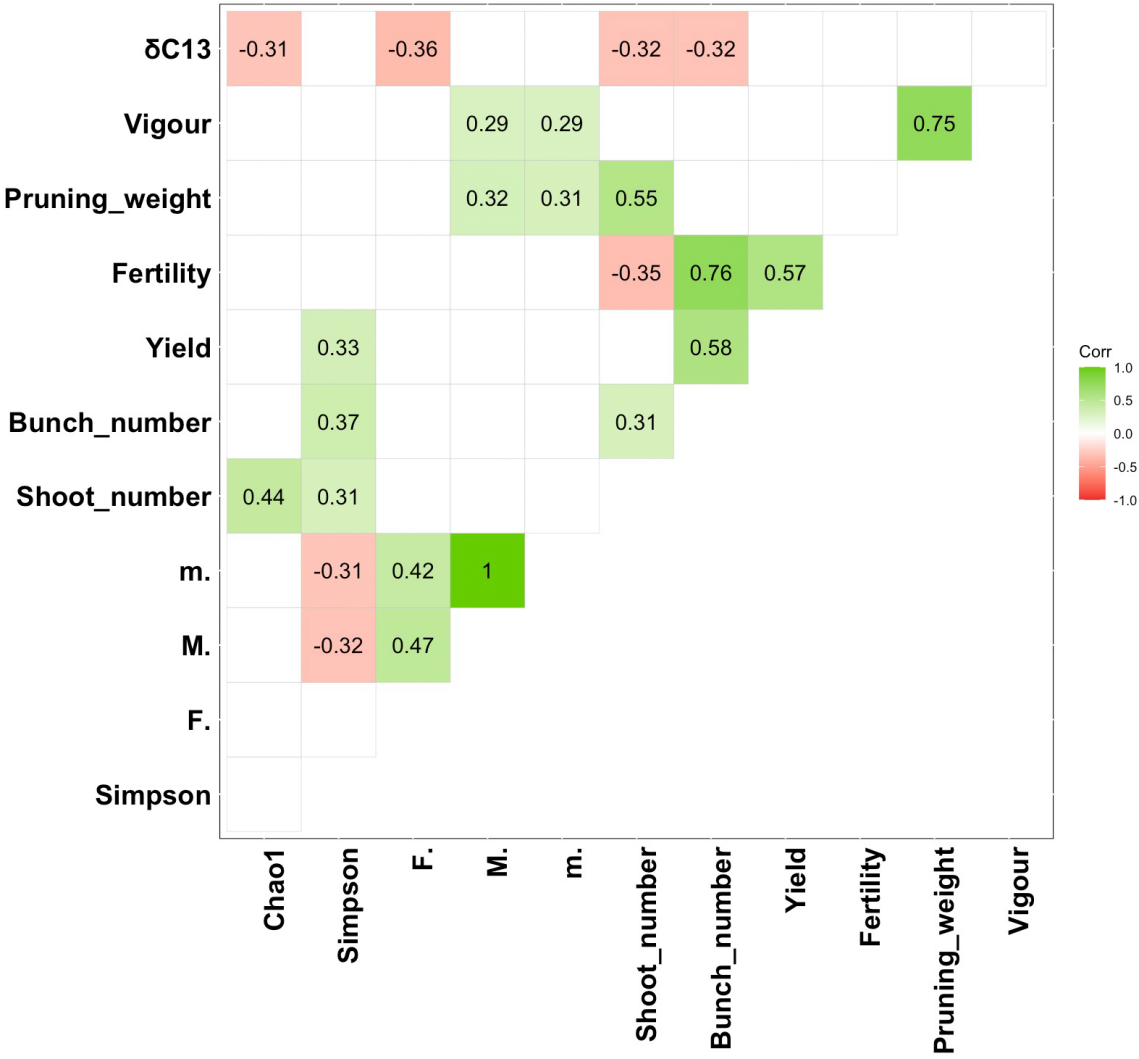
Matrix showing significant Pearson correlations between AMF communities related to microbial variables and plant phenotypic traits measured among the 10 rootstock × scion combinations (*p* value <0.05).

AMF variables assessed by microscopy and sequencing of the 18S rRNA gene in the 10 rootstock × scion combinations were correlated with plant phenotypic traits. Ten significant Person's correlations (*p* values <0.05) were established between AMF variables and plant phenotypic traits, ranging from −0.36 to 0.44 (Figure 5). The richness (Chao1) correlated negatively with the δ^13^C (*r* = −0.31) and positively with shoot number (*r* = 0.44). The diversity (Simpson) correlated with yield (*r* = 0.33), number of bunches (*r* = 0.37), and number of shoots (*r* = 0.31), suggesting that the lower the diversity, the higher the yield and the number of bunches and shoots. The mycorrhization frequency (F%) was negatively correlated with the δ^13^C (*r* = −0.31). Finally, the overall mycorrhization intensity (M) and the mycorrhization intensity of mycorrhized fragments (m%) were correlated with the vigour (*r* = 0.29), and the pruning weight (*r* = 0.32 and *r* = 0.31, respectively). These correlations highlight the link between AMF community of the root system and grapevine growth.

### Comparison between 18S and 28S rRNA genes sequencing to study AMF communities

**FIGURE 6:**
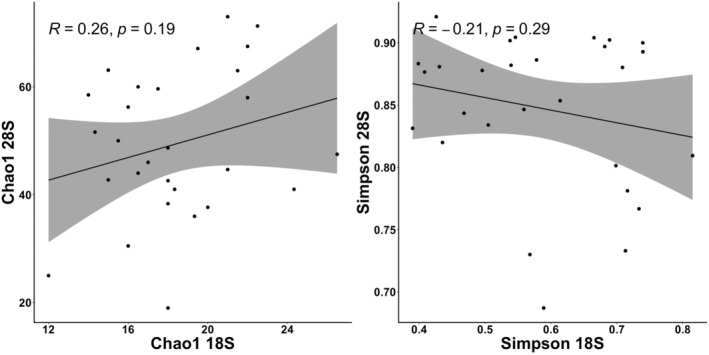
Pearson correlations of α‐diversity metrics measured by sequencing of 18S and 28S rRNA genes.

Using the 28S rRNA gene of glomeromycotans, 170 OTUs were successfully affiliated with three orders (*Diversisporales*, *Glomerales*, and *Paraglomerales*), six families (*Acaulosporaceae*, *Claroideoglomeraceae*, *Diversisporaceae*, *Gigasporaceae*, *Glomeraceae*, and *Paraglomeraceae*), eight genera and 30 species (Table S7). With the 18S rRNA gene, 72 OTUs were successfully affiliated with the same orders and families as above but subdivided into seven genera and 25 species (Table S8). Abundance graph showed similar relative abundances of AMF genera between methodologies (Figures 2A and 3A). However, 9% of total sequences were classified as “multi‐affiliated *Glomeraceae*” with the 28S rRNA gene. The genera *Rhizophagus* was detected with the 28S rRNA gene only representing <1% of total sequences. Both approaches showed an effect of the rootstock genotype on the Bray–Curtis index. However, the significant difference between SO4 and 3309C was repeated with both methodologies, whereas the significant difference between 1103P and SO4 was only observed with the 28S rRNA gene. No significant Pearson correlation was found between α‐diversity metrics measured by sequencing of 18S and 28S rRNA genes (Figure 6). Among the different rootstocks, LEfSe detected the enrichment of 18 and 20 taxa with the 18S and the 28S rRNA gene, respectively. Although certain enrichments of order, families and genera were observed with both techniques, others were not, especially at the species level. When the correlation matrices between α‐diversity metrics and plant phenotypic traits measured in the six rootstocks grafted with CS were assessed with the 18S rRNA gene, Chao1 index was significantly correlated (*p* value <0.05) with the shoots number (*r* = 0.44), the fertility (*r* = −0.54) and the δ^13^C (*r* = −0.38), but Simpson index did not correlate with any phenotypic traits (Figure S3A). When the correlations were established with α‐diversity metrics measured with the 18S rRNA methodology on the same individuals, the Chao1 index was significantly correlated (*p* value <0.05) with the vigour (*r* = −0.38), whereas the Simpson index was correlated with the δ^13^C (*r* = −0.38) (Figure S3B). In addition, the α‐diversity metrics obtained with the 28S rRNA gene were significantly correlated together (*r* = 0.58).

## DISCUSSION

### The rootstock and scion genotypes do not influence the mycorrhization capacity of the root system

AMF structures in the roots have long been analysed by staining with trypan blue (Gaši et al., 2023; Landi et al., 2021). This product is classified as carcinogenic (H350) and presents risks for the operator. For this reason, we used the Shaeffer black ink as a safe alternative which is effective in staining AM structures (Duret et al., 2022; Vierheilig et al., 1998). The arbuscular mycorrhiza colonization rate was evaluated using the method of Trouvelot et al. (1986). No effect of the rootstock or the scion genotypes was observed on the frequency of mycorrhization (F%) which was close to 100% in all the individuals tested. Landi et al. (2021) detected the same frequency among three vineyards in central‐eastern Italy. Taken together, these results suggested that grapevine associate with indigenous populations of AMF in the soil, raising questions about the inoculation process of mycorrhizal fungi in grapevine. Our overall mycorrhization intensity was 42% with values ranging from 14% to 81%, which is consistent with study by Landi et al. (2021). A similar result was obtained for the mycorrhization intensity of mycorrhized fragments $\left(m=\frac{M\times 100}{F}\right)$ because *F* was closed to 100% in all rootstock genotypes. Although the arbuscular abundance (A%) provided by the Mycocalc program is an interesting parameter to study AMF communities, we decided not to take it into account. This was due to the fact that when the score of the semi‐quantitative scale used to quantify the mycorrhization reaches 4 or 5, the large number of hyphae and vesicles prevents accurate observation of the arbuscules for quantification. These microscopic analyses suggested that the choice of rootstock and scion genotype did not influence the mycorrhization capacity of the root system. The significant differences observed by Darriaut et al. (2022) between RGM and 1103P rootstocks grown in asymptomatic soil were not repeated here. Our results are consistent with those of Sportes et al. (2023), who reported that rootstock varieties did not affect mycorrhizal colonization when grown in a greenhouse for 1 month. However, Fors et al. (2023) showed a significant effect of the rootstock on the root mycorrhizal colonization by comparing 110R and 1103P. These differences can be explained by the conditions under which the plants were grown, and the microscopy method used, which varied between experiments.

### Metabarcoding analyses of the 28S rRNA gene showed that rootstock genotype influences the AMF communities of the root system

In this study, analyses were carried out on the root system of vines grown on block 1 of the GreffAdapt plot. Among the diversity metrics measured with the 28S rRNA methodology, rootstock genotype significantly influenced the Bray–Curtis index only. The AMF community of SO4 was significantly distinct from those of 1103P and 3309C. Several AMF taxa were enriched in the root system of the six rootstock genotypes. In a previous study, we carried out the same type of analysis on different samples, collected in May 2021, from the same rootstock genotypes grafted with CS in blocks 2 and 3 of the same plot (Lailheugue et al., 2024). Although an effect of the rootstock genotype was reported on the Bray–Curtis index in both rhizosphere and root endosphere compartments, significant differences between rootstock genotypes were observed exclusively in the rhizosphere. In this previous study, the rootstock genotype influenced both Chao1 and Simpson metrics, but only three taxa were enriched in 1103P and Nem rootstocks (Lailheugue et al., 2024). These differences between experiments could partly be explained by the season of sampling. We hypothesize that the season may have an impact on various environmental and physiological factors of the plant, and consequently on its rhizodeposition in terms of biochemical composition, as reviewed by Ma et al. (2022). The nature of plant rhizodeposition (i.e., root exudate, mucilage, root border cells, dead root cap cells) is known to influence the microbiome of the root system, including AMF communities (Philippot et al., 2013). However, this hypothesis is in contradiction with the results of Schreiner and Mihara (2009) who observed similar AMF phylotypes between the phenological stages of bloom and véraison in Pinot Noir. Although the soil analyses were relatively similar between blocks, differences were found in the structure of block 1, such as a higher percentage of coarse elements and a lower one of fine soil. It is already known that soil properties influence AMF diversity (Darriaut et al., 2023; Schreiner & Mihara, 2009). Although certain results differed between the two experiments, which may be due to the characteristics of the soil and the sampling season, the rootstock genotype had a significant impact on the AMF community of the root endosphere.

### Assessment of the AMF diversity by 18S rRNA gene sequencing confirmed an influence of the rootstock but presented little or no contribution of the scion

As for the gene encoding the 28S rRNA gene, the metabarcoding analysis of the 18S rRNA gene showed an effect of the rootstock genotype on the Bray–Curtis index which was significantly different between SO4 and 3309C. In addition, this analysis detected an effect of the genotype on the Chao1 index which was significantly higher in 1103P compared to 41B. Finally, several taxa of AMF were enriched in the roots of the six rootstock genotypes with the 18S rRNA gene, which were or were not in common with those observed with the 28S rRNA gene. Although differences were observed between the results of the two approaches, the use of two target genes confirmed that the rootstock genotype drives the AMF communities of the root system in grapevine, as proposed in our previous study (Lailheugue et al., 2024). The differences observed could be explained by the choice of the primers amplifying different regions which can affect the detection of some fungal species (Suzuki et al., 2020), as well as the use of the nested PCR targeting the 28S rRNA gene, which could result in a decrease of AMF taxa and diversity (Yu et al., 2015). The lack of consensus for the sequencing of AMF communities makes it very difficult to achieve meta‐analysis. Our results are consistent with those of Moukarzel et al. (2021) who were the first authors to demonstrate that rootstock genotype determines the root‐colonizing AMF community, based on the trap culture analysis by DGGE of grapevine roots from three vineyards and nine rootstocks. Other studies carried out on other grafted fruit trees revealed an influence of the rootstock genotype on the AMF composition of the root system of lemon (Song et al., 2020) and apple trees (van Horn et al., 2021). The plant genotype is also a factor affecting the rhizodeposition that explains the differences of root AMF community between rootstock genotypes (Ma et al., 2022). The composition of certain root exudates has already been shown to vary between rootstock genotypes such as organic acids (Cançado et al., 2015), amino‐acids (Marastoni et al., 2020) and strigolactones (Lailheugue et al., 2023).

Concerning the effect of the scion, similar proportions of AMF genera were observed in the root system of RGM grafted with the five scions. None of the cultivars were significantly different in pairwise Adonis comparisons, although an effect of the scion genotype was reported on the Bray–Curtis index. Finally, no effect of the scion was reported on α‐diversity metrics and few enriched taxa were detected in the roots of RGM grafted with the different cultivars. These results were consistent with those obtained in May 2021, using 28S rRNA methodology, which showed no effect of the scion genotype on both ⍺‐ and β‐diversity metrics measured in the roots and the rhizosphere (Lailheugue et al., 2024). Although the scion is responsible for the production and allocation of sugars to the rootstock (Ollat et al., 2017), our experiments revealed that its genotype has little or no influence on the AMF communities associated with the root system of the same rootstock. Interestingly, Song et al. (2020) reported no effect of the scion genotype on several α‐diversity metrics in citrus. However, PCoA based on the OTUs abundance indicated that the composition of AMF communities was mainly affected by the rootstock genotype rather than the scion.

### The AMF community of the root system provides benefits to grapevine

Several correlations were found between AMF variables and plant phenotypic traits. The *M* (%) and *m* (%) correlated positively to vegetative growth‐related traits (vigour and pruning weight), as well as the Chao1 and Simpson indexes which correlated to the number of shoots. Previous studies reported that AMF inoculation improves the growth of grapevine aerial parts (Moukarzel et al., 2023; Nicolás et al., 2015). In the current study, the F (%) and the AMF richness (Chao1) correlated negatively to δ^13^C, suggesting either that mycorrhizal symbiosis is more significant when the water status is less constraining, or that the intensity of the water stress is lower when plants are more mycorrhized. This last hypothesis is consistent with another study showing that AMF are able to improve grapevine water status and alleviate drought stress (Torres et al., 2021). AMF diversity (Simpson) correlated with the plant productivity, indicating that yield and number of bunches are higher when diversity is low. Although no studies have investigated the effect of the AMF diversity on grapevine yields, several reports proposed that grapevine inoculation with AMF improves it (Torres et al., 2018; Trouvelot et al., 2015). Interestingly, the AMF diversity was negatively correlated to *m* (%) and *M* (%), suggesting that competition between species occurs during the colonization of the root system, as proposed by Moukarzel et al. (2023). In a previous study, no significant correlations were found between plant phenotype and ⍺‐diversity metrics measured for AMF in the roots harvested in May 2021 (Lailheugue et al., 2024). This may be explained by the differences in soil properties between blocks or the choice of the target genes, as we observed herein by comparing the correlation matrices obtained with the 18S rRNA and the 28S rRNA.

## CONCLUSION

This study revealed that the colonization of grapevine root systems by AMF occurs independently of both rootstock and scion genotypes. However, we demonstrated with two sequencing methods (i.e., 28S and 18S rRNA genes) that different rootstock genotypes, subjected to the same pedoclimatic conditions, drive the AMF community of the root system. The choice of methodology to analyse AMF diversity had an impact on the metabarcoding results, emphasizing the need for a consensus strategy to explore AMF composition using DNA‐based approaches. Analyses of the 28S rRNA gene confirmed that the scion genotype makes little or no contribution to the AMF diversity of the root system. Finally, several parameters measured to quantify the AMF communities in the root system using microscopy and metabarcoding approaches were correlated with the plant phenotype, highlighting the beneficial roles of AMF in grapevine development and adaptation to environmental conditions.

## AUTHOR CONTRIBUTIONS

**Vincent Lailheugue:** Investigation; data curation; methodology; formal analysis; visualization; writing – original draft; writing – review and editing. **Romain Darriaut:** Investigation; data curation; formal analysis; methodology; writing – review and editing. **Joseph Tran:** Data curation; formal analysis; methodology; visualization; writing – review and editing. **Marine Morel:** Investigation; formal analysis; writing – review and editing. **Elisa Marguerit:** Conceptualization; investigation; writing – review and editing. **Virginie Lauvergeat:** Conceptualization; funding acquisition; project administration; supervision; writing – original draft; writing – review and editing.

## CONFLICT OF INTEREST STATEMENT

The authors declare no conflicts of interest.

## Supporting information


**DATA S1.** Supporting Information.

**TABLE S7.** Affiliation of the 170 OTU obtained after sequencing of the 28S rRNA gene.

**TABLE S8.** Affiliation of the 72 OTU obtained after sequencing of the 18S rRNA gene.

## Data Availability

All raw sequencing data is available on the European Nucleotide Archive (ENA) database under accession number PRJEB71926 and secondary accession number ERP156713.
