# Supplementary material for: The rootstock modifies the arbuscular mycorrhizal community of the root system, while the influence of the scion is limited in grapevines
Source: Environ Microbiol Rep. 2024 Aug 22;16(4):e13318. doi: 10.1111/1758-2229.13318 (PMC11340015; doi:10.1111/1758-2229.13318)
Supplement: Supplementary file 1 — DATA S1. Supporting Information. [file EMI4-16-e13318-s002.pdf]

Supplemental Data

Supplemental Figures

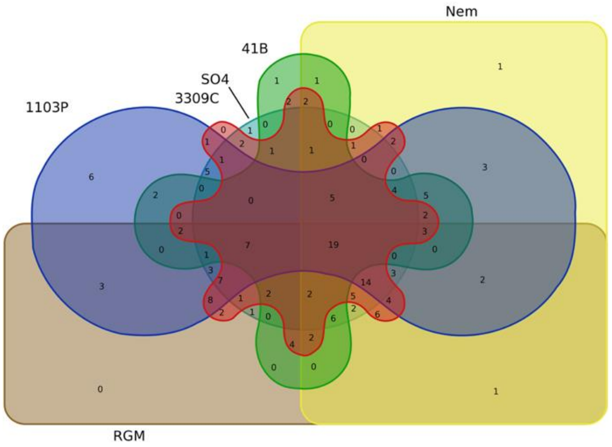

Figure S1: Venn diagram showing common and exclusives OTUs observed with the 28S rRNA gene between rootstock genotypes.

A

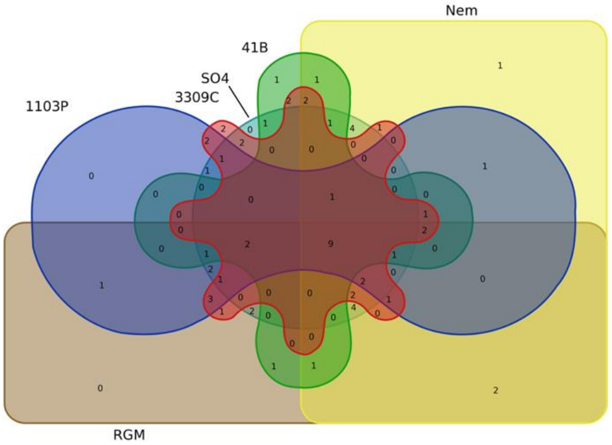

B

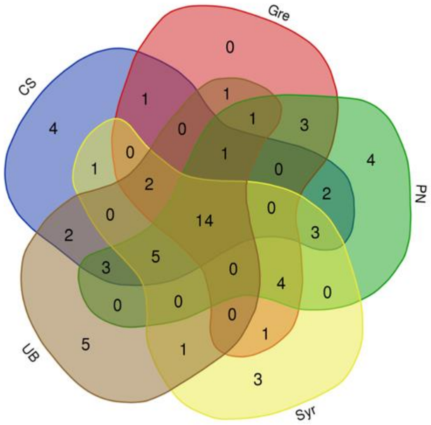

Figure S2: Venn diagram displaying common and exclusives OTUs observed with the 18S rRNA gene among rootstock (A) and scion (B) genotypes.

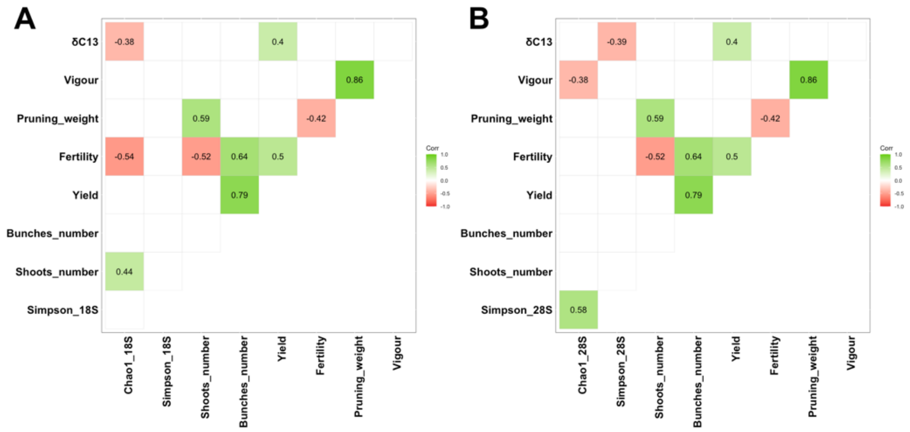

Figure S3: Matrix showing significant Pearson correlations (*P*-value < 0.05) of the variables to assess AMF community and plant phenotypic traits measured in the 5 rootstocks grafted with CS with the 18S (A) and the 28S (B) rRNA genes approaches.

Supplemental Tables

Table S1: Soil analysis carried out on the sampled block of the GreffAdapt plot.

	**Block 1**
**Depth (cm)**	0-35
**Coarse elements (%)**	47.6
**Fine soil (%)**	52.4
**Coarse sands (‰)**	725
**Fine sands (‰)**	110
**Coarse silts (‰)**	14
**Fine silts (‰)**	73
**Clay (‰)**	79
**Texture given**	Sand
**Organic matter (%)**	2.3
**Total nitrogen (%)**	0.103
**C / N**	12.8
**Water pH**	6.5
**Absorbent complex**	
**Potassium (g / 100g)**	0.71
**Magnesium (g / 100g)**	1.29
**Calcium (g / 100g)**	16
**C.E.C. (cmol+ / kg)**	6.2
**Saturation (%)**	105.3

Table S2: Rootstocks and scion cultivars used in this study.

	Name	Genetic backgrounds	Characteristics	
			Conferred vigour	Water deficit tolerance
**Rootstocks grafted with Cabernet Sauvignon**	Selection Oppenheim 4 (SO4)	*V. berlandieri* × *V. riparia*	High	Medium to good
	3309 Couderc (3309C)	*V. riparia* × *V. rupestris*	Low to medium	Weak in top soil
	41 B MGt (41B)	*V. berlandieri* × *V. vinifera*	Medium to high	Medium to high
	1103 Paulsen (1103P)	*V. berlandieri* × *V. rupestris*	High	Good
	Riparia Gloire de Montpellier (RGM)	*V. riparia*	Very low to low	Weak
	Nemadex AB	*V. berlandieri* × *V. Muscadinia* × *V. vinifera* × *V. rupestris*	Low	Weak
**Scion cultivars grafted on RGM**			Berry color	
	Cabernet-Sauvignon clone 169	*V. vinifera*	Red	
	Syrah clone 524	*V. vinifera*	Red	
	Grenache clone 362	*V. vinifera*	Red	
	Pinot noir clone 113	*V. vinifera*	Red	
	Ugni blanc clone 481	*V. vinifera*	White	

Table S3: List of primers and PCR conditions used for the metabarcoding approaches.

Target gene	Primer	Sequence (5'-3')	Initial denaturation	Number of cycles	Denaturation	Annealing	Extension	Final extension	Reference
**Eurayote 28S rRNA**	LR1	GCATATCAATAAGCGGAGGA	95°C 5min	30	95°C 30s	58°C 30s	72°C 30s	72°C 5min	van Tuinen et al., 1998
	NDL22	TGGTCCGTGTTTCAAGACG							
**AMF 28S rRNA**	FLR3	TTGAAAGGGAAACGATTGAAGT	98°C 30s	30	95°C 30s	55°C 30s	72°C 45s	72°C 7min	Gollotte et al. 2004
	FLR4	TACGTCAACATCCTTAACGAA							
**AMF 18S rRNA**	AMV4.5NF	AAGCTCGTAGTTGAATTTCG	95°C 5min	35	95°C 45s	58°C 45s	72°C 1min	72°C 7min	Sato et al. 2005
	AMDGR	CCCAACTATCCCTATTAATCAT							
**Illumina adapters**	Forward	TCGTCGGCAGCGTCAGATGTGTATAAGAGACAG							PGTB
	Reverse	GTCTCGTGGGCTCGGAGATGTGTATAAGAGACAG							

Table S4: Comparison of the Bray-Curtis index between rootstock genotypes grafted with CS assessed by sequencing of 28S rRNA gene.

pairs	Df	SumsOfSqs	F.Model	R2	p.value	p.adjusted	sig
RGM vs 1103P	1	0.696	2.803	0.259	0.017	0.255	ns
RGM vs Nem	1	0.412	2.054	0.204	0.06	0.9	ns
RGM vs SO4	1	0.527	2.548	0.242	0.032	0.48	ns
RGM vs 41B	1	0.984	4.460	0.389	0.005	0.075	ns
RGM vs 3309C	1	0.392	1.923	0.194	0.107	1	ns
1103P vs Nem	1	0.802	3.864	0.326	0.009	0.135	ns
**1103P vs SO4**	**1**	**1.101**	**5.143**	**0.391**	**0.003**	**0.045**	*****
1103P vs 41B	1	0.881	3.849	0.355	0.068	1	ns
1103P vs 3309C	1	0.795	3.768	0.320	0.012	0.18	ns
Nem vs SO4	1	0.208	1.253	0.135	0.348	1	ns
Nem vs 41B	1	1.075	6.181	0.469	0.024	0.36	ns
Nem vs 3309C	1	0.942	5.781	0.419	0.008	0.12	ns
SO4 vs 41B	1	1.191	6.563	0.484	0.011	0.165	ns
**SO4 vs 3309C**	**1**	**1.288**	**7.603**	**0.487**	**0.003**	**0.045**	*****
41B vs 3309C	1	0.777	4.367	0.384	0.032	0.48	ns

Table S5: Comparison of the Bray-Curtis index between rootstock genotypes grafted with CS assessed by sequencing of 18S rRNA gene.

pairs	Df	SumsOfSqs	F.Model	R2	p.value	p.adjusted	sig
RGM vs 1103P	1	0.242	1.263	0.153	0.284	1	ns
RGM vs Nem	1	0.316	1.757	0.180	0.064	0.96	ns
RGM vs SO4	1	0.539	2.547	0.242	0.063	0.945	ns
RGM vs 41B	1	1.000	4.838	0.377	0.016	0.24	ns
RGM vs 3309C	1	0.154	1.295	0.139	0.294	1	ns
1103P vs Nem	1	0.331	1.564	0.183	0.267	1	ns
1103P vs SO4	1	0.510	2.055	0.227	0.124	1	ns
1103P vs 41B	1	0.693	2.861	0.290	0.063	0.945	ns
1103P vs 3309C	1	0.328	2.315	0.249	0.056	0.84	ns
Nem vs SO4	1	0.258	1.125	0.123	0.391	1	ns
Nem vs 41B	1	0.827	3.691	0.316	0.019	0.285	ns
Nem vs 3309C	1	0.485	3.569	0.309	0.005	0.075	ns
SO4 vs 41B	1	0.842	3.286	0.291	0.024	0.36	ns
**SO4 vs 3309C**	**1**	**0.930**	**5.529**	**0.409**	**0.003**	**0.045**	*****
41B vs 3309C	1	0.993	6.083	0.432	0.027	0.405	ns

Table S6: Comparison of the Bray-Curtis index between scion genotypes grafted onto RGM assessed by sequencing of 18S rRNA gene.

pairs	Df	SumsOfSqs	F.Model	R2	p.value	p.adjusted	sig
UB vs Syr	1	0.502015	2.700824	0.252394	0.114	1	ns
UB vs Gre	1	0.556977	4.709322	0.370541	0.037	0.37	ns
UB vs CS	1	0.269643	1.700544	0.175304	0.175	1	ns
UB vs PN	1	0.39121	2.320988	0.22488	0.15	1	ns
Syr vs Gre	1	0.584027	3.910598	0.328329	0.06	0.6	ns
Syr vs CS	1	0.496845	2.619989	0.246704	0.055	0.55	ns
Syr vs PN	1	0.42204	2.114148	0.209029	0.116	1	ns
Gre vs CS	1	0.16415	1.345126	0.143939	0.315	1	ns
Gre vs PN	1	0.15257	1.155629	0.126221	0.293	1	ns
CS vs PN	1	0.094659	0.54934	0.064255	0.568	1	ns
